# Assessing the feasibility of the transmastoid infralabyrinthine approach without decompression of the jugular bulb to the extradural part of the petrous apex and petroclival junction prior to surgery

**DOI:** 10.1007/s00701-024-06044-8

**Published:** 2024-03-26

**Authors:** Zafer Cinibulak, Jörg Poggenborg, Stefanie Schliwa, Shadi Al-Afif, Nima Ostovar, Joachim K. Krauss, Makoto Nakamura

**Affiliations:** 1 Department of Neurosurgery, Merheim Hospital, Ostmerheimer Str. 200, 51109 Cologne, Germany; 2 Department of Radiology, Merheim Hospital, Cologne, Germany; 3 Faculty of Health, Herdecke University, WittenWitten, Germany; 4 Institute of Anatomy, Anatomy and Cell Biology, University of Bonn, Bonn, Germany; 5 Department of Neurosurgery, Hannover Medical School, Hannover, Germany

**Keywords:** Petrous apex, Petroclival junction, Infralabyrinthine approach, Neuronavigation, Facial nerve, Jugular bulb

## Abstract

**Background and objective:**

This study aims to define specific measurements on cranial high-resolution computed tomography (HRCT) images prior to surgery to prove the feasibility of the navigated transmastoid infralabyrinthine approach (TI-A) without rerouting of the facial nerve (FN) and decompression of the jugular bulb (JB) in accessing the extradural-intrapetrous part of petrous bone lesions located at the petrous apex and petroclival junction.

**Materials and methods:**

Vertical and horizontal distances of the infralabyrinthine space were measured on cranial HRCT images prior to dissection. Subsequently, the area of access was measured on dissected human cadaveric specimens. Infralabyrinthine access to the extradural part of the petrous apex and petroclival junction was evaluated on dissected specimens by two independent raters. Finally, the vertical and horizontal distances were correlated with the area of access.

**Results:**

Fourteen human cadaveric specimens were dissected bilaterally. In 54% of cases, the two independent raters determined appropriate access to the petrous apex and petroclival junction. A highly significant positive correlation (*r* = 0.99) was observed between the areas of access and the vertical distances. Vertical distances above 5.2 mm were considered to permit suitable infralabyrinthine access to the extradural area of the petrous apex and petroclival junction.

**Conclusions:**

Prior to surgery, vertical infralabyrinthine distances on HRCT images above 5.2 mm provide suitable infralabyrinthine access to lesions located extradurally at the petrous apex and petroclival junction via the TI-A without rerouting of the FN and without decompression of the JB.

## Introduction

Petrous apex (PA) and petroclival lesions can vary in their pathology [1, 2, 6, 7, 10, 11, 27, 32, 38]. They can be extradural, intradural, or transdural. The origin, growth direction, size, and extent of these lesions play a crucial role in deciding the surgical approach.

Lesions originating intradurally predominately extend within the intradural space. They rarely extend extradurally. For these lesions, intradural transcranial middle fossa and posterior fossa approaches are usually selected [16, 18, 23, 31, 41].

Lesions originating extradurally are mostly located intraosseous. These lesions regularly invade the petrous bone, locally enclose important intrapetrous neurovascular structures, and grow in different directions. While merely displacing the petroclival dura and respecting or violating minimally the dural integrity, these lesions usually extend extradurally into the posterior fossa, middle fossa, jugular foramen, or cavernous sinus [4, 11]. Given their intraosseous growth pattern, they can reach sizable dimensions, promote bone erosion, and displace neurovascular structures before causing neurologic symptoms that will eventually lead to diagnosis [11].

In most cases, the goal is gross- or near-total removal [27, 35, 38]. However, in certain cases, gross- or near-total resection can pose a significant risk of morbidity and mortality. In such instances, only subtotal removal is performed. Postoperatively, depending on the histological examination, these patients can be managed with radiotherapy or regular radiologic follow-up [11, 27].

Due to the sizable dimension of these extradural lesions at the time of diagnosis, a single- or multiple-stage procedure with different approaches is necessary to achieve the surgical goal while minimizing neurologic deficits. The transmastoid infralabyrinthine approach (TI-A) without rerouting of the facial nerve (FN) has been described as a feasible and safe approach for the part of these lesions located extradurally at the PA and petroclival junction (PJ) [12, 13, 26].

Usually, to increase the infralabyrinthine extradural access to the PA and PJ, the jugular bulb (JB) is skeletonized and slightly compressed downwards [26]. However, skeletonizing of the JB carries the risk of bulb injury, which can lead to significant bleeding and air embolism and may trigger thrombosis, brain edema, or venous infarction through compression of the JB [33]. To overcome these risks, it is possible to leave the JB bone covered with a thin lamella of bone in cases the position of the JB provides sufficient infralabyrinthine space.

Currently, there is a lack of detailed studies assessing the feasibility of the neuronavigated TI-A without rerouting the FN and leaving the JB covered with a thin lamella of bone for accessing the extradural part of the PA and the petroclival region.

This study aims to evaluate radiological measurements of the infralabyrinthine space on high-resolution computed tomography (HRCT) images before surgery to determine the applicability of this approach.

## Material and methods

### Cadaver characteristics and surgical tools

Prior to and after dissection, a cranial HRCT (Siemens SOMATOM Sensation 64, Erlangen, Germany) was obtained in all human head specimens. The images were acquired using a petrosal bone scan protocol (120 kV, 200 mAs, collimation 12 mm × 0.6 mm, pitch 0.8, CTDIvol 46.5 mGy (16 cm)) and reconstructed in a bone window (u90).

Mastoidectomy and petrosectomy, leaving the JB covered with a thin lamella of bone, were performed neuronavigated (Medtronic StealthStation S7 System, Louisville, USA) under a neurosurgical microscope (Leica M500N OHS-1, Heerbrugg, Switzerland) by drilling the bone with a high-speed drill (Aesculap Elan-E, Germany) and using standard microsurgical instruments.

**Fig. 1:**
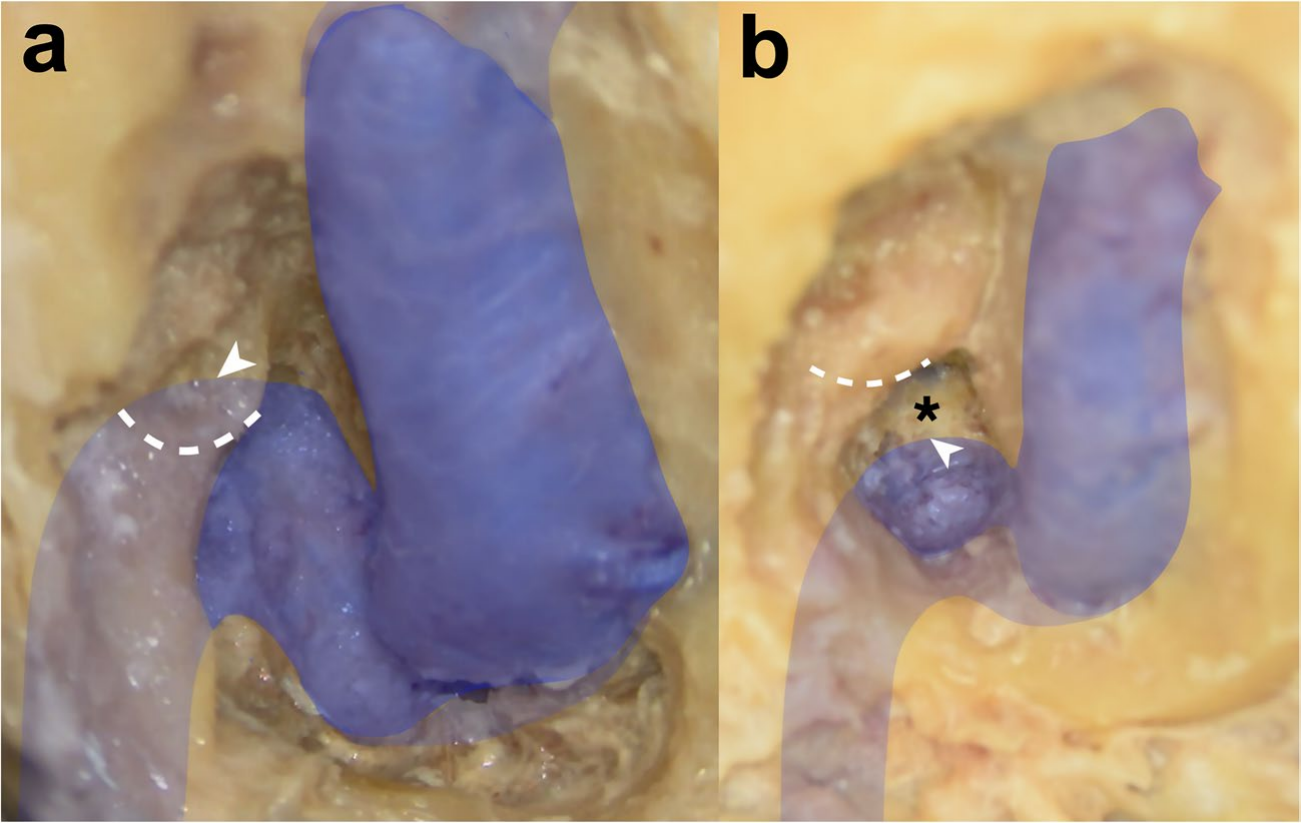
Classification of the infralabyrinthine area based on the position of the jugular bulb **a** The position of the apex (white arrowhead) of the jugular bulb (marked with blue color) reaches over or to the roof of the posterior semicircular canal (dashed white line) (type A). There is no infralabyrinthine area between the jugular bulb and the posterior semicircular canal. **b** The apex of the jugular bulb (white arrowhead) does not reach the lower edge of the posterior semicircular canal (dashed white line) (type B). An infralabyrinthine area (black asterisk) between the jugular bulb and the posterior semicircular canal is available

Depending on the radiological vertical extension, the infralabyrinthine area was initially classified into two groups: type A with the apex of the JB reaching over or to the roof of the posterior semicircular canal (PSCC) (no infralabyrinthine space between the PSCC and the JB) (Fig. 1a) and type B with the apex of the JB not reaching the lower edge of the PSCC (available infralabyrinthine space between the PSCC and the JB). Dissection was only carried out on type B classified infralabyrinthine bones (Fig. 1b).

### Dissection technique

The cadaver head was fixed in a Mayfield clamp and rotated 45° to the opposite side. The Medtronic navigation system was set up, and the reference arm was secured to the Mayfield clamp. Throughout the measurement course, the relationship between the reference arm and the cadaver head remained constant.

**Fig. 2:**
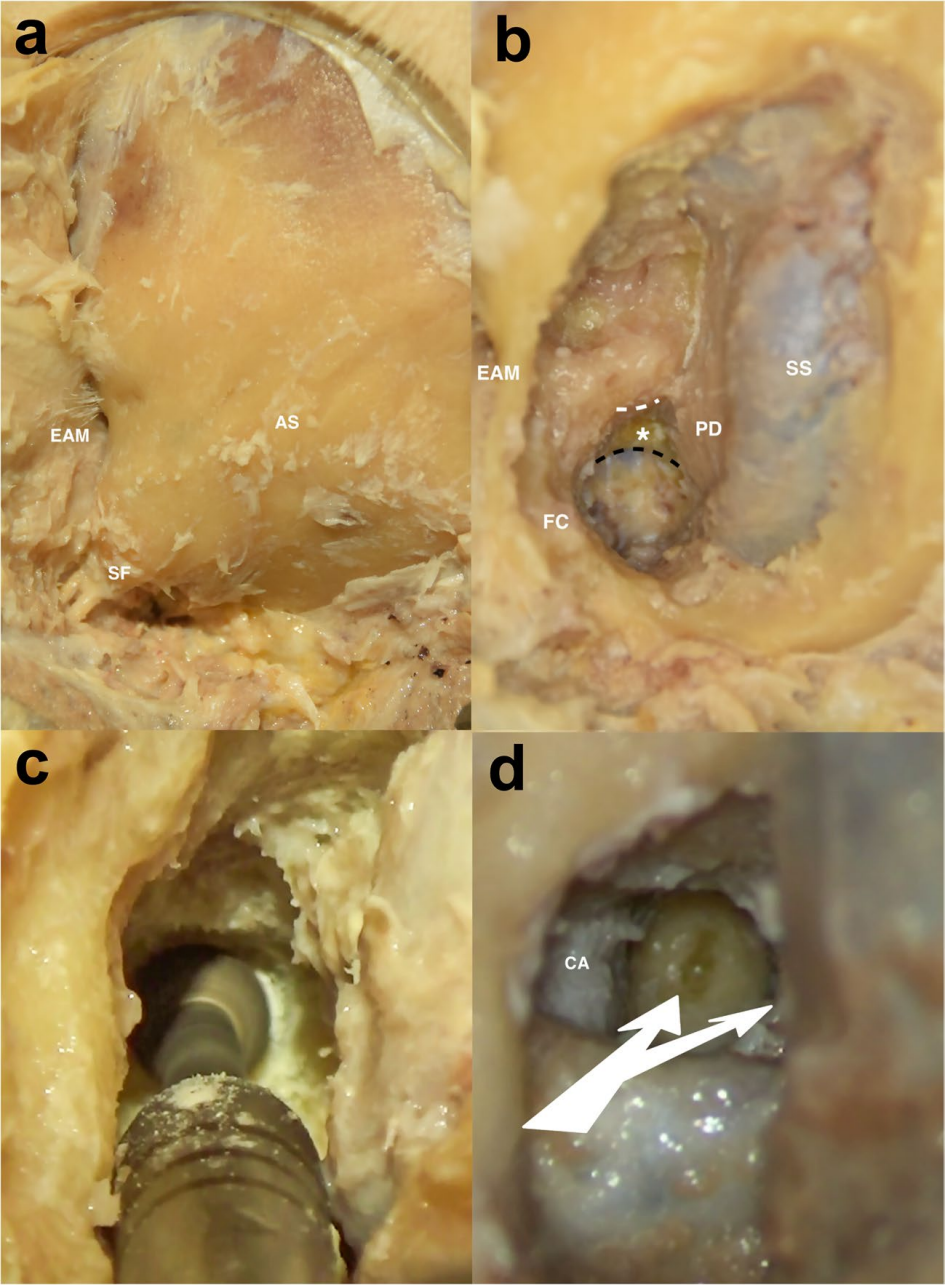
Dissection technique of the neuronavigated transmastoid infralabyrinthine approach without rerouting the facial nerve and with bone-covered jugular bulb **a** Mastoid bone with exposed asterion (AS), styloid foramen (SF), and external acoustic meatus (EAM). **b** Mastoid bone removed between the sigmoid sinus (SS), the presigmoidal dura (PD), the fallopian canal (FC), and the external acoustic meatus (EAM) with revealing of the infralabyrinthine area (white asterisk) below the lower edge of the posterior semicircular canal (dashed white line) and above the apex of the jugular bulb (covered with a thin bone lamella; dashed black line). **c** Drilling of the petrous bone through the infralabyrinthine window with a diamond burr while preserving the surrounding anatomical structures. **d** Access to the petrous apex and petroclival junction (split white arrow) through an infralabyrinthine and suprabulbar route. The medial surface of the carotid artery (CA) is visualized

A retroauricular curvilinear c-shaped skin incision was made, and the skin flap was reflected ventrally to expose the complete mastoid bone (Fig. 2a). Subsequently, under continual utilization of the neuronavigation, a mastoidectomy was performed until the sigmoid sinus with the presigmoidal dura and the fallopian canal were visualized. Gradually removing the mastoid bone ventromedial to the fallopian canal and ventrolateral to the presigmoidal dura allowed the exposure of both the osseous PSCC and the JB (Fig. 2b).

Infralabyrinthine access to the PA and PJ was obtained by drilling the bone between the osseous PSCC and the JB (Fig. 2c). The fallopian canal and the osseous PSCC were preserved. The JB was left covered with a thin bone lamella (Fig. 2d).

### Measurements

The following measurements were determined: the vertical distance, the horizontal distance, and the area of access.

**Fig. 3:**
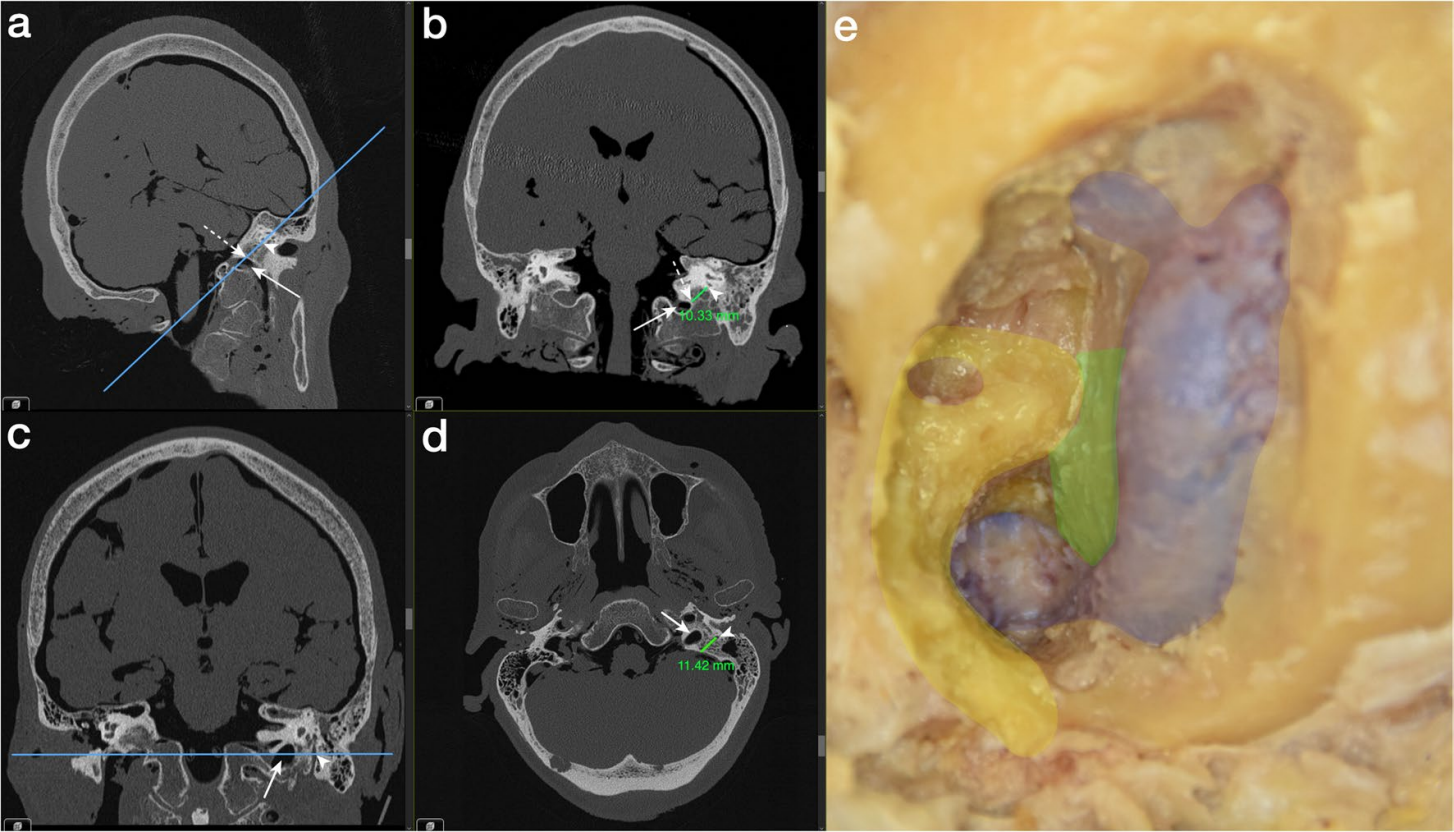
Annotated radiological measurements before dissection and anatomical overview of the infralabyrinthine access window on the left side **a** Reformatted and tilted sagittal and **b** cranial high-resolution computed tomography (CT) scans transecting the lower edge of the bony posterior semicircular canal (white arrowhead), the jugular bulb (white arrow), and the apex of the jugular bulb (dotted white arrow) are highlighted. The cutline (blue line) on the tilted sagittal scan overlies the minimal infralabyrinthine distance between the bony posterior semicircular canal and the apex of the jugular bulb, denoting the plane of the coronal image. The measured vertical distance (10.33 mm) is demonstrated on the coronal image. **c** Reformatted and tilted coronal and **d** axial cranial high-resolution CT scans transecting the jugular bulb (white arrow) and the midpoint of the fallopian canal (white arrowhead) are displayed. The cutline (blue line) on the tilted coronal scan, intersecting the midpoint of the fallopian canal, indicates the axial cut plane. The measured horizontal distance (10.33 mm) running parallel to the trajectory of the jugular bulb and extending between the midpoint of the fallopian canal and the presigmoidal dura is shown on the axial image. **e** Color marked overview of the infralabyrinthine access window with preserved osseous posterior semicircular canal and fallopian canal (yellow), sigmoid sinus and bone-covered jugular bulb (blue), and presigmoidal dura (green)

The vertical distance corresponds to the shortest distance between the lower border of the osseous PSCC and the apex of the JB covered with a thin bone lamella (Fig. 3a, b), and the horizontal distance corresponds to the distance between the midpoint of the fallopian canal and the presigmoidal dura, running parallel to the trajectory of the jugular bulb (Fig. 3c, d).

The vertical and horizontal distances were measured on reformatted and tilted HRCT images before dissection. Radiologic measurements were performed in a bone window with Vue PACS (Phillips, Stuttgart, Germany) using the source images on the workstation.

The area of access is defined as the infralabyrinthine area between the osseous PSCC and the JB. This area has approximately a quadrangular form, bounded by four fixed anatomical points: the medial border of the fallopian canal at the second genu, the medial border of the osseous PSCC, the superior border of the most accessible distal part of the JB, and the presigmoidal dura at the entry point of the LCNs IX–XI (Fig. 3e).

The measurements related to the area of access were obtained neuronavigated on the dissected human head specimens. The *x*, *y* and *z* coordinates were collected holding the stereotactic probe on the defined fixed target points. All coordinates were computed into an Excel spreadsheet (Microsoft Office Excel 2013, Microsoft Corp.).

To calculate the quadrangle area of access based on the coordinates of its four corner points, the quadrangle exposed area was divided into two triangle areas with the corner points forming the triangle vertices. Each triangle area (*T*∆) was calculated via Heron’s formula (Weisstein):

$$T\Delta = \frac{1}{2}\times \sqrt{{(X1-X2)}^{2}+{(Y1-Y2)}^{2}+{(Z1-Z2)}^{2}}\times \sqrt{{(X1-X3)}^{2}+{(Y1-Y3)}^{2}+{(Z1-Z3)}^{2}}$$

Then, both corresponding triangle areas were added together to get the area of access.

### Approval of the surgical access

**Fig. 4:**
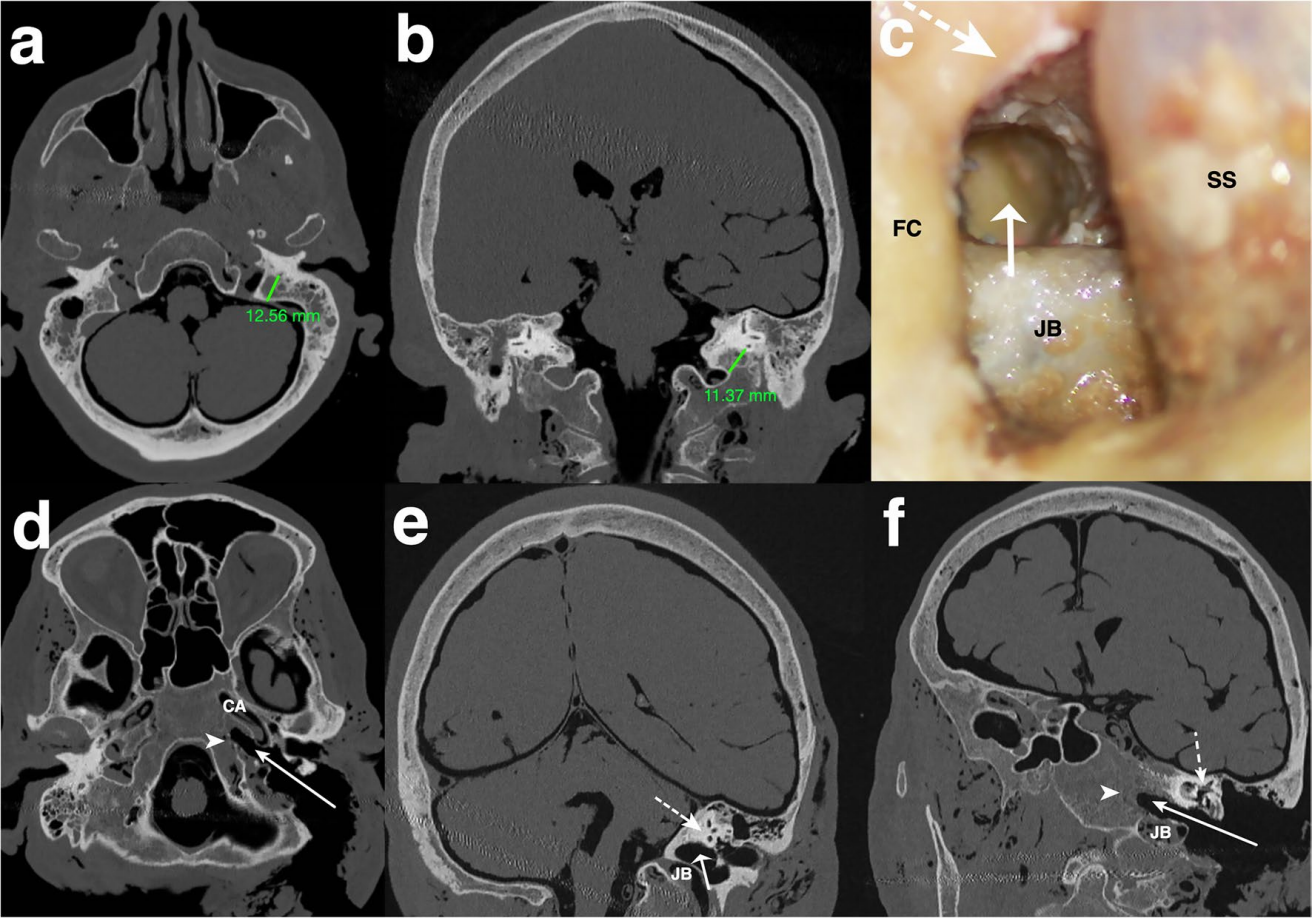
Suitable categorized infralabyrinthine access path on the left side **a** Axial and **b** coronal cranial high-resolution CT scan with measurements of the horizontal distance (12.56 mm) and vertical distance (11.37 mm) before dissection. Overview of the successful carried out TI-A (**c**) on a dissected human head specimen, along with reformatted and tilted **d** axial, **e** coronal, and **f** sagittal high-resolution cranial CT scans. The delineated access route (white arrow), running just below the intact osseous labyrinth (dotted white arrow) and medial to the horizontal segment of the carotid artery (CA) to reach the segment of the petrous apex (PA) and petroclival junction (PJ) (white arrowhead) within the lower petrous region, is illustrated. The sigmoid sinus (SS) is skeletonized, the fallopian canal (FC) is preserved, and the jugular bulb (JB) is left within a thin shell of bone

**Fig. 5:**
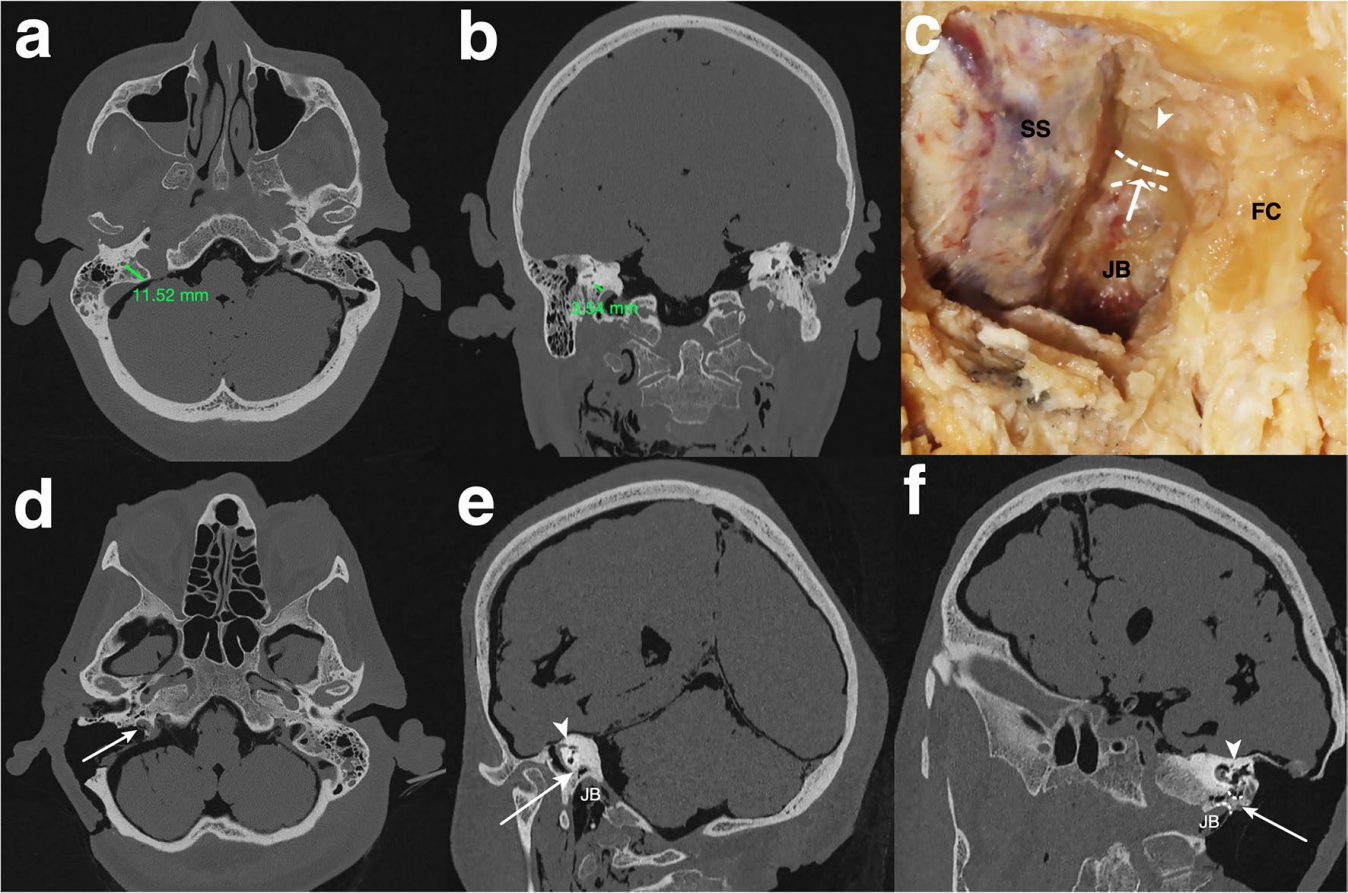
Not suitable categorized infralabyrinthine access path on the right side **a** Axial and **b** coronal high-resolution cranial computed tomography (CT) scans display the horizontal (11.52 mm) and vertical (3.54 mm) distance prior to dissection in the respective planes. Illustrating of an unsuitable area of access to the petrous apex (PA) and petroclival junction (PJ) (**c**) on a dissected human head specimen, and on reformatted and tilted **d** axial, **e** coronal, and **f** sagittal high-resolution cranial CT scans. A limited infralabyrinthine suprabulbar space (area between the two dotted lines), indicating an inadequate access route (white arrow) to the PA and PJ via the TI-A, is outlined. The sigmoid sinus is skeletonized, and the fallopian canal (FC) and osseous labyrinth (white arrowhead) are preserved, with the jugular bulb (JB) left covered by a thin shell of bone

Infralabyrinthine access was categorized by two independent authors (ZC, NO) for each side. The approach was categorized as suitable (Y) only if the extradural bone of the PA and at the PJ could be removed through the gained infralabyrinthine window and confirmed on the HRCT images after dissection (Fig. 4a–f). Otherwise, it was categorized as not suitable (N) (Fig. 5a–f). Surgical access was approved for each side if both authors confirmed the approach was suitable.

### Statistical analysis

Statistical analysis of the calculated values was performed using SPSS (IBM SPSS Statistics for Mac, version 23.0. Armonk, NY, USA), including the mean value, standard deviation, maximum and minimum values, range, and variance.

An unpaired *t* test was conducted to compare the mean values of vertical distance, horizontal distance, and the area of access between the not suitable and suitable groups.

Agreement between the two raters was analyzed with the kappa statistic.

In order to establish a correlation between the presurgical radiological measurements and rated surgical access via the neuronavigated TI-A without rerouting of the FN and with the JB covered with a thin lamella of bone, two correlations were determined with the Pearson correlation coefficient (*R*) between 1 (the area of access with the vertical distance) and 2 (the area of access with the horizontal distance). A positive *R* value indicates a positive correlation, and a negative *R* value indicates a negative correlation. *R* values less than 0.5 or − 0.5 were considered weak relationships, and *R* values greater than 0.8 or − 0.8 were regarded as a very high. All other *R* values were considered to indicate a high correlation.

Values of *p* < 0.05 were considered statistically significant.

## Results

Twenty-eight sides of 14 fixed human cadaver heads were dissected in the skull base laboratory of Merheim Hospital. All values were carried out successfully for each side and documented in tabular form (Table 1). Distances and areas were calculated to one decimal place.

**Table 1:** Rating of the approach suitability related to the radiological and anatomical measured values

Type	Side	Radiological measurements before dissection		Anatomical measurements on dissected specimens	Rater	
		Vertical distance (mm)	Horizontal distance (mm)	Area of access window (mm^2^)	ZC	NO
Type A	Right	0	10.5	0	N	N
	Left	0	11.4	0	N	N
	Right	0	11.8	0	N	N
	Right	0	12.3	0	N	N
	Right	0	11.6	0	N	N
	Right	0	10.6	0	N	N
	Left	0	12.4	0	N	N
	Right	0	11.7	0	N	N
	Left	2.1	10.7	31.1	N	N
	Right	2.4	12.1	23.7	N	N
	Right	2.8	11.9	28.4	N	N
	Right	3.5	11.5	30.1	N	N
	Right	4.3	12.1	39.6	N	Y
Type B	Left	2.1	10.7	31.1	N	N
	Right	2.4	12.1	23.7	N	N
	Right	2.8	11.9	28.4	N	N
	Right	3.5	11.5	30.1	N	N
	Right	4.3	12.1	39.6	N	Y
	*Left*	*5.2*	*11.9*	*51.7*	*Y*	*Y*
	*Left*	*5.4*	*12.2*	*51.9*	*Y*	*Y*
	*Right*	*6.1*	*12.7*	*58.4*	*Y*	*Y*
	*Left*	*8.2*	*12.3*	*86.5*	*Y*	*Y*
	*Right*	*8.5*	*12.4*	*80.4*	*Y*	*Y*
	*Left*	*8.7*	*12.4*	*84.1*	*Y*	*Y*
	*Left*	*10.3*	*11.4*	*97.2*	*Y*	*Y*
	*Left*	*11.4*	*12.6*	*122.4*	*Y*	*Y*
	*Right*	*11.4*	*13.1*	*121.7*	*Y*	*Y*
	*Right*	*11.5*	*12.6*	*130.1*	*Y*	*Y*
	*Left*	*12.6*	*11.5*	*125.1*	*Y*	*Y*
	*Left*	*12.7*	*12.4*	*126.0*	*Y*	*Y*
	*Left*	*12.8*	*11.7*	*129.9*	*Y*	*Y*
	*Left*	*12.9*	*12.4*	*125.4*	*Y*	*Y*
	*Left*	*13.4*	*12.8*	*131.1*	*Y*	*Y*

The values were documented tabularly in descending order according to the measured vertical distance. Suitable rated approaches by both raters are presented in italics. The values for the vertical and horizontal distances are given in millimeters (mm), and the values for the access window are in millimeters squared (mm^2^)

### Vertical and horizontal distance

The measured vertical and horizontal distances before dissection allow the surgeon to estimate the vertical and horizontal maneuverability at the infralabyrinthine area through which the instruments will be introduced to reach the petrous bone.

The calculated mean value for the vertical distance was in total 5.9 ± SD 5.1 mm (not suitable group, 1.2 ± SD 1.6 mm; suitable group, 10.1 ± SD 2.9 mm; unpaired *t* test between the not suitable and suitable groups, *p* < 0.01), and that for the horizontal distance was in total 12.1 ± SD 0.7 mm (not suitable group, 11.6 ± SD 0.6 mm; suitable group, 12.3 ± SD 0.5 mm; unpaired *t* test between the not suitable and suitable groups, *p* < 0.01). For the total vertical distance, a large range with up to 13.4 mm (not suitable group, up to 4.3 mm; suitable group, up to 8.2 mm) was observed, and for the horizontal distance, a small range with up to 2.6 mm (not suitable group, up to 1.9 mm; suitable group, up to 1.6 mm) was observed (Table 2). The large range for the vertical distance may be due to the high variability in size, shape, and position of the JB.

**Table 2:** Mean values of measurements

Surgical access	Vertical distance (mm)						Horizontal distance (mm)						Access window (mm^2^)					
	Min	Max	Mean	SD	Var	*p*	Min	Max	Mean	SD	Var	*p*	Min	Max	Mean	SD	Var	*p*
Not suitable	0	4.3	1.2	1.6	2.6	< 0.01	10.5	12.4	11.6	0.6	0.4	< 0.01	0	39.6	11.8	15.9	251.3	< 0.01
Suitable	5.2	13.4	10.1	2.9	8.1		11.5	13.1	12.3	0.5	0.2		51.7	131.1	101.5	30.3	919.1	
Total	0	13.4	5.9	5.1	25.8		10.6	13.1	12.0	0.7	0.4		0	131.1	59.8	51.6	2662.8	

The values for the vertical distance, horizontal distance, and access window are shown for not suitable rated access, suitable rated access, and in total. The *p* values of the unpaired *t* test, comparing the mean values of the vertical distances, horizontal distances, and access windows between the non-suitable and suitable groups, are also displayed. The measured vertical and horizontal distances are presented in millimeters (mm), and the values for the access windows are presented in millimeters squared (mm^2^)

*Max* maximum, *Min* minimum, *SD* standard deviation, *Var* variance

### Area of access

The area of access was defined as the entrance to the target region in the surgical field. It corresponds to the infralabyrinthine bone between the fallopian canal, the posterior SCC, and the JB. Greater vertical and horizontal extensions enlarge this area and may facilitate surgical access.

The mean value for the area of access in total was calculated at 59.8 ± SD 51.6 mm^2^ (not suitable group, 11.8 mm^2^; suitable group, 101.5 mm^2^; unpaired *t* test between the not suitable and suitable groups, *p* < 0.01), with a large range up to 131.1 mm^2^ (not suitable group, up to 39.6 mm^2^; suitable group, up to 79.4 mm^2^) (Table 2).

There was a 95.8% agreement on the categorized surgical access with a kappa value of 0.864 (*p* < 0.01) between the two raters.

On 15 sides (54%), the 2 raters categorized the surgical access to the petrous bone as appropriate (Table 1).

**Fig. 6:**
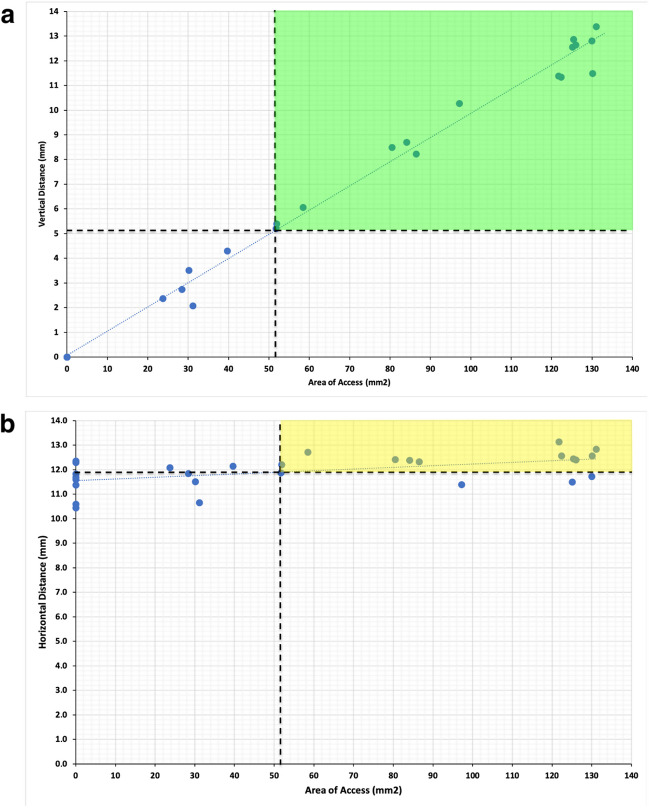
Correlation of the area of access with the vertical and horizontal distances.The correlation of the area of access with **a** the vertical distance and **b** the horizontal distance is illustrated. A very high positive correlation is demonstrated for the vertical distance and a weak negative correlation for the horizontal distance. The highlighted green area represents the gained sufficient access to the petroclival junction (PJ) and petrous apex (PA) for the vertical distance and the yellow area for the horizontal distance

A very high positive and statistically significant correlation (*R* = 0.99; *p* < 0.01) was observed between the vertical distance and the area of access (Fig. 6a), and a weak negative correlation (*R* =  − 0.39; *p* < 0.05) was observed between the horizontal distance and the area of access (Fig. 6b).

The correlations indicate that the vertical distance primarily influences the extent of the access window. According to the area values categorized as suitable by the two independent raters (area values > 51.7 mm^2^) and the correlations of the areas of access with the vertical and horizontal distances, vertical distances above 5.2 mm were categorized/deemed as suitable by the two independent neurosurgeons.

### Illustrative case

**Fig. 7:**
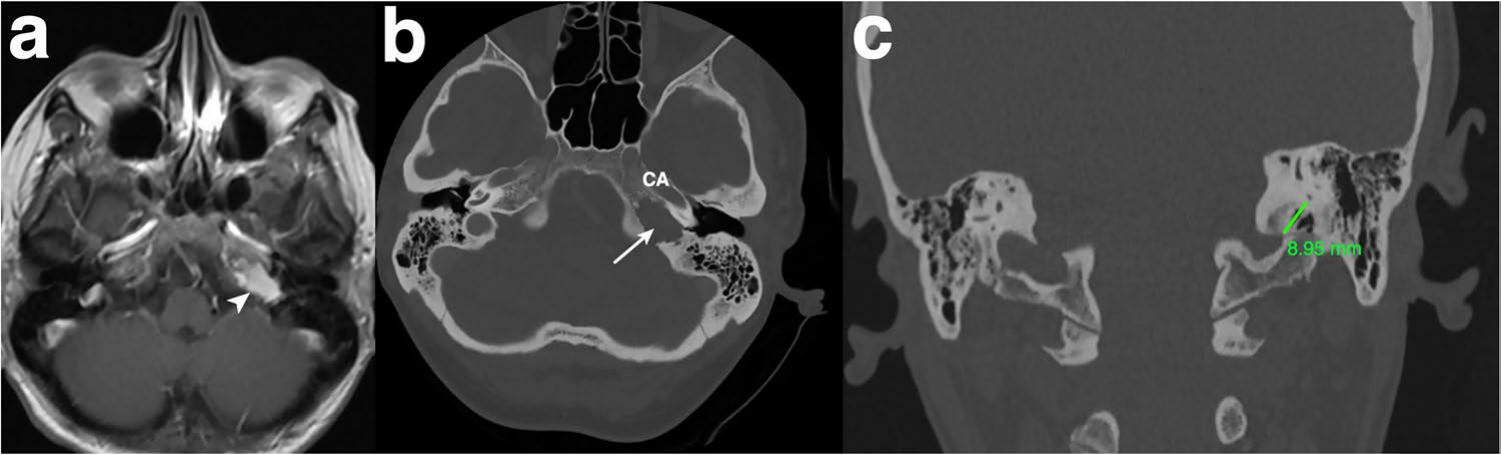
Evaluation of the feasibility of the TI-A for accessing an intrapetrous extradural lesion at the petrous apex (PA) and petroclival junction (PJ) **a** Preoperative T1-weighted axial contrast-enhanced magnetic resonance imaging (MRI) and **b** axial high-resolution bone window computed tomography (CT) scan are presented, showing a contrast-enhancing lesion (white arrowhead) with osseous erosion (white arrow). **c** The measured horizontal distance of 8.95 mm on the coronal cranial CT indicates adequate infralabyrinthine access

A 48-year-old woman presented with a history of headache, dizziness, and dysphagia. Cranial magnetic resonance imaging (MRI) with gadolinium administration (Fig. 7a) and cranial computed tomography (CT) (Fig. 7b) showed a 22 mm × 20 mm × 26 mm contrast-enhancing osteolytic lesion within the left petrous bone. The lesion was localized infralabyrinthine with extension to the dorsal part of the horizontal petrous portion of the carotid canal and close to the ventral border of the jugular bulb. No involvement of the petrous apex, destruction of the internal auditory canal (IAC), and the osseous labyrinthine could be detected.

**Fig. 8:**
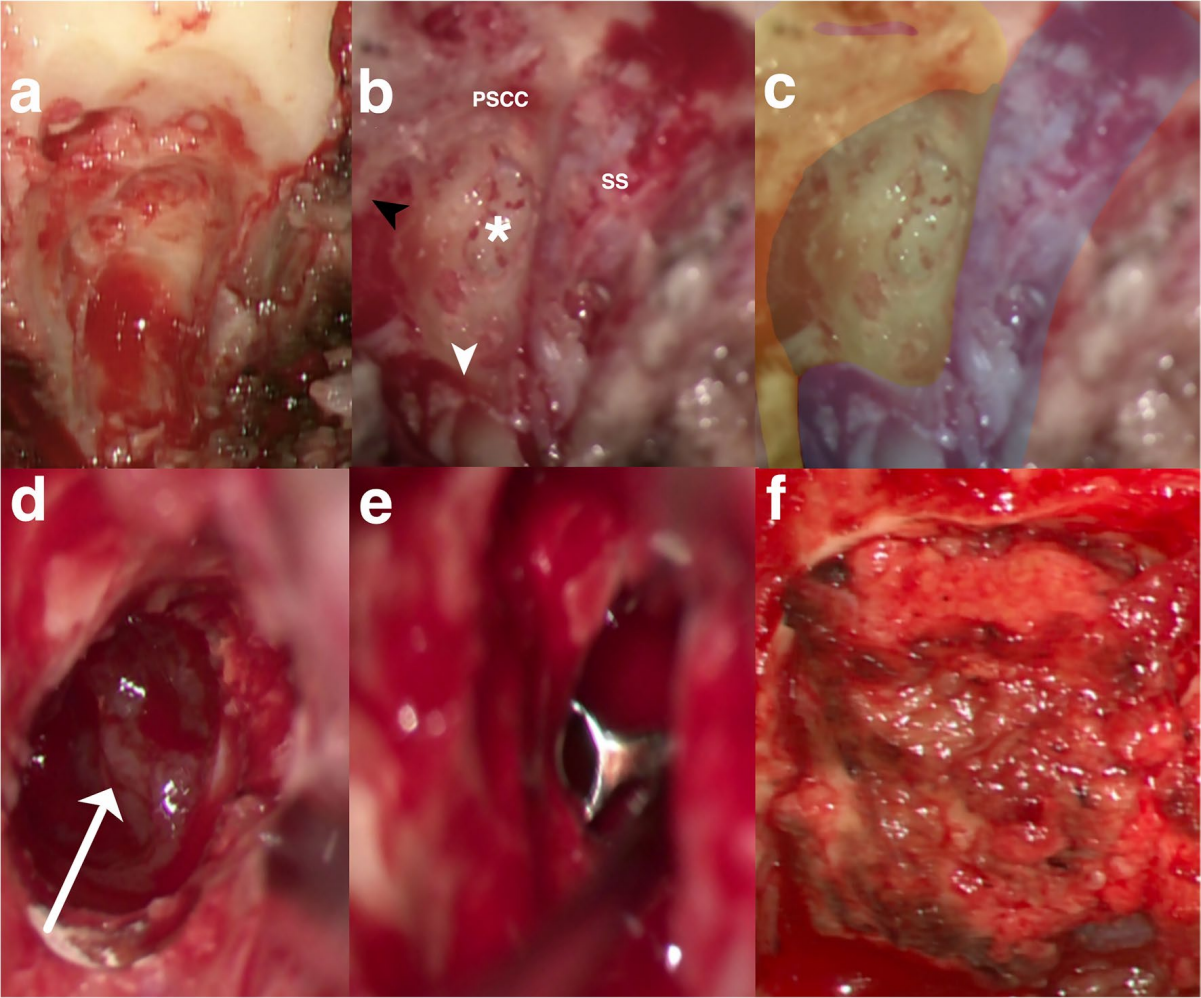
Stepwise microsurgical exposure of the TI-A without rerouting the facial nerve and with bone-covered JB to the part of the petrous apex (PA) and petroclival junction (PJ) within the lower petrous portion.**a** Presigmoidal retrofacial mastoidectomy and surgical access window with surrounding anatomical structures on the left side are **b** shown in situ and **c** highlighted with colors. The access window infralabyrinthine suprabulbar (white star, marked green), the preserved fallopian canal (black arrowhead, marked yellow) with the posterior semicircular canal (PSCC), and the jugular bulb (JB, marked blue) left bone covered with a thin shell of bone (white arrowhead) are demonstrated. **d** Visualization of the dorsal part of the tumor (white arrow) and **e** tumor resection with curette after drilling of the infralabyrinthine suprabulbar bone. **f** Reconstruction of the skull defect with a small muscle placed on the residual tumor cavity sealed with fibrin glue

Preoperatively, on the high-resolution cranial CT, the measured value for the vertical distance was noted to be above 5.2 mm on the left side (Fig. 7c). According to our anatomical findings, the neuronavigated TI-A without rerouting the FN and without skeletonizing the JB was stated as suitable. Resection was performed successfully (Fig. 8a–f).

**Fig. 9:**
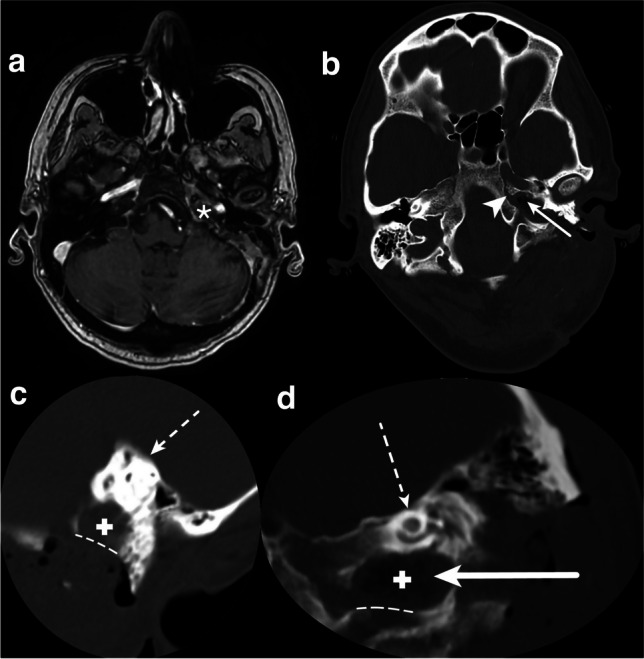
Postoperative cranial magnetic resonance imaging (MRI) and computed tomography (CT) confirming tumor removal via the TI-A **a** Postoperative axial MRI after gadolinium administration demonstrating no contrast-enhancing tumor mass (white star). Reformatted and tilted **b** axial, **c** coronal, and **d** sagittal cranial CT reveal access to the tumor cavity (white cross), located beneath the inferior part of the extradural petroclival junction (PJ) (white arrowhead), via an osseous infralabyrinthine approach pathway (white arrow). The jugular bulb covered by a thin shell of bone (white dotted line) and the preserved bony labyrinth (dotted white arrow) are visualized

The postsurgical course was uneventful without any neurological deteriorations. Dysphagia as well as dizziness improved completely. Postoperative MRI and CT (Fig. 9a–d) confirmed total resection. Histology subsequently reported WHO grade II chondrosarcoma.

## Discussion

The present cadaveric study and the illustrative case emphasize that the neuronavigated TI-A without rerouting the FN and without skeletonizing the JB permits a safe route to drain, biopsy, and remove extradural lesions in the PA and PJ. The feasibility of the TI-A by leaving the JB covered with a thin layer of bone depends primarily on the position of the JB. Based on the coronal HRCT images, suitable surgical access was deemed possible for vertical distances above defined values between the apex of the JB and the PSCC.

Petroclival and PA lesions can be classified into intradural, extradural, or transdural lesions. They can be of different origin [1, 2, 6, 8, 10, 11, 27, 32, 38].

Lesions originating extradurally at the PJ or PA are usually soft tissue masses that regularly invade the petrous bone and locally encase important intrapetrous neurovascular structures [27]. Respecting dural integrity, these lesions can expand into the posterior fossa, middle fossa, jugular foramen, or cavernous sinus while merely displacing the dura [4]. Due to the slow growth pattern, symptoms typically appear late. Therefore, they are often large at diagnosis [11]. The predominantly extradural spreading and the soft tissue consistency of these lesions make them exceptionally well suited for surgical removal through extradural approaches to the petrous bone.

Since no single approach can provide access to all extradural areas of the petrous bone, a single-stage or multiple-stage procedure using various approaches can be necessary to achieve the surgical goal of a gross- or near-total resection while minimizing neurological deficits [24, 36, 40]. In cases where performing a gross- or near-total resection poses a high risk of morbidity and mortality, the possibility of a subtotal resection can be considered. Postoperative management of the extradural lesions depends on the histological findings and may involve radiotherapy or regular radiologic follow-up [11, 27].

Combinations of approaches with the lowest risk of morbidity and mortality can be selected based on achieving the surgical goal of a gross- or near-total resection while considering the location of the lesions, tissue consistency, and comorbidity of the patient [21].

Combinations of the following surgical approaches with different routes have been described for addressing these lesions: suboccipital retrosigmoid approach (posterior route), extradural subtemporal approach (anterolateral route), endoscopic transnasal approach (anteromedial approach route), and TI-A without rerouting of the FN (lateral route).

The retrosigmoid approach is safe and relatively easy to perform. It provides an excellent view of the intradural PA and petroclival region. Therefore, this approach is suitable for removing the portion that has extended to the posterior fossa [19, 34]. For the intrapetrous located part, the retrosigmoid approach is unsuitable because the middle and lower cranial nerves and IAC are situated directly within the path of the approach, hindering access.

The extradural subtemporal approach is an entirely extradural approach that overcomes excessive retraction on the temporal lobe. Access to the PA and PJ is gained through two rhomboid petrous bone areas, the outer rhomboid area (Kawase quadrangle) and the inner rhomboid area (fenestrated rhomboid area).

The outer rhomboid area is delineated by the petrous ridge, V3 branch of the trigeminal nerve, greater superficial petrosal nerve, and arcuate eminence. This area, with an average size of 269.13 mm^2^ [39], provides wide exposure to the upper petrous portion (part of the intrapetrous bone, PA and PJ, extending from the arcuate eminence up to the trigeminal impression located superior to the horizontal segment of the intrapetrous carotid artery, Dorello’s canal, and IAC) [7, 25].

Equally, the inner rhomboid area serves as the entrance to the inferior portion of the rostral PJ. It is enclosed by the IAC dorsally, the horizontal part of the ICA laterally, the dura of the posterior fossa medially, and the accessible ventral part of the intrapetrous bone just medial to the horizontal ICA anteriorly. Despite a larger average size of 64.5 mm^2^ [3] compared to our calculated access area, the angled surgical perspective and constrained maneuverability of instruments, induced by the positioning of the temporal lobe, make it unsuitable for accessing the lower petrous portion (part of the intrapetrous bone, PA and PJ, extending inferior to the horizontal segment of the intrapetrous carotid artery, Dorello’s canal, and IAC).

Thus, this approach is appropriate for accessing extradural-originated lesions located within the upper petrous region, especially centered in the petrous apex near the superior petrosal sinus or in the superior portion of the petrous apex. However, accessing the lower petrous portion and the infralabyrinthine area dorsal to the IAC is restricted [17].

Endoscopic endonasal approaches to the petrous bone are safe and effective. Access is gained through an anteromedial, well-defined triangular bone corridor (Gardner’s triangle). The sides of this triangular bone corridor are defined by the paraclival internal carotid artery anterolaterally, the abducens nerve posteromedially, and the petroclival synchondrosis inferiorly. Compared to our approach, the mean area of access has been described as significantly higher, with 87.56 ± SD 20.06 mm^2^ [15]. However, this approach provides surgical access only to the tip of the PA. Endoscopic access to the intrapetrous area is restricted due to the position of the ICA.

This limitation is particularly relevant for pathologies localized within the intrapetrous region without extending to the tip of the PA or the paraclival and lacerum segments of the ICA medially. To effectively approach these pathologies, it is necessary to enhance the triangular access corridor.

An expanded endoscopic endonasal corridor has been described involving the transecting of the synchondrosis of the foramen lacerum, disconnecting the lacerum fibrous tissue from the Eustachian tube cartilage, or resecting the Eustachian tube, and mobilizing the paraclival and lacerum ICA superiorly and laterally [9]. The expanded corridor allows for additional access to the intrapetrous dorsocaudal and dorsomedial portions including the PJ. Access to the upper petrous portion is not possible.

If the lesion extends medially towards the paraclival and lacerum segments of the ICA, resulting in superior and lateral displacement of the ICA, a naturally expanded triangular access corridor will be created. This corridor facilitates the resection of the lesion without requiring further enlargement of the access pathway.

Therefore, endoscopic approaches are described as ideal for lesions adjoining the sphenoid sinus and/or the paraclival and lacerum segments of the ICA medially [20, 21, 28].

For pathologies localized within the lower petrous region without pathology-related expansion of the triangular access corridor, the decision of the approach, either TI-A without rerouting of the FN and without skeletonizing of the JB or endoscopic endonasal approach, depends on the proximity of the pathology to the respective areas of access. Infralabyrinthine-located part of the lesions situated dorsal to the IAC have already been described as inappropriate for endoscopic endonasal approaches [17, 20, 28].

For the extradural part of the lesions located within the PA and along the entire PJ, the IT-A without rerouting of the FN has been described as a feasible and safe approach [26]. If the position of the JB induces an obstacle, the JB can be skeletonized and slightly compressed with bone wax or neurosurgical patties to increase infralabyrinthine access [14, 26].

Skeletonizing the JB, however, bears the risk of bulb injury [22]. This can induce brisk venous bleeding or air embolism. Also, compression on the JB can trigger subsequent bulb thrombosis [33].

Therefore, in cases where the initial position of the JB permits sufficient surgical access to the extradural part of the PJ and PA, skeletonizing the JB can be avoided. This was also supported by Miller et al. [26]. In their cohort of four patients presenting PA and petroclival lesions with minimal to no intracranial extension, they noted in one case that the position of the JB permitted sufficient infralabyrinthine corridor allowing the removal without skeletonizing of the JB.

The present study examined vertical and horizontal distances of the infralabyrinthine access window on HRCT images before dissection to quantify accurately which measured values of the dissected infralabyrinthine corridor without skeletonizing of the JB and without rerouting of the FN permit sufficient access to remove PA and petroclival lesions located within the lower petrous portion. While the vertical distance plays an important role in determining the feasibility of the IL-A without skeletonizing the JB for accessing the PA and the PJ, the horizontal distance plays a minor role. Adequate surgical access was established for infralabyrinthine vertical dimensions greater than 5.2 mm. This allows surgeons to obtain tissue for histological examination and remove the extradural-intrapetrous located part of PA and petroclival lesions.

Additional use of computer-assisted navigation facilitates obtaining an optimal approach corridor while preserving the anatomical integrity and allowing safe exposure [5, 29, 30, 37].

The primary limitation is that this study is based on cadaveric dissections only. Clinical studies with large patient numbers have to be conducted to verify the applicability of this approach according to the measured values. Additionally, the measurements on the HRCT images before dissection may have been overestimated or underestimated compared to the real distances on the dissected specimens.

## Conclusion

The TI-A without rerouting the FN and without skeletonizing the JB provides an effective route to the part of the PA and PJ located within the lower petrous portion. For infralabyrinthine vertical distances above 5.2 mm, the feasibility of this approach can be depicted radiologically on HRCT images before surgery. Leaving the JB covered with bone reduces the risk of bulb injury and bulb thrombosis. Using the neuronavigation allows safe exposure and facilitates obtaining the optimal approach corridor while preserving anatomical integrity. The TI-A, leaving the JB covered with a thin bone lamella, can be considered for approach combination to remove the part of extradural originated lesions that have extended to the part of the PA and PJ within the lower petrous portion

## Data Availability

Data available within the article.

## Abbreviations

FN: Facial nerve
HRCT: High-resolution computed tomography
IAC: Internal auditory canal
JB: Jugular bulb
PA: Petrous apex
PJ: Petroclival junction
PSCC: Posterior semicircular canal
TI-A: Transmastoid infralabyrinthine approach

## Declarations

### Additional information

The present work was accepted for oral presentation at the 3rd International Rhoton Society (3rd IRS) Meeting in Istanbul, Turkey, in August 2023.
